# Predicting prognosis and clinical efficacy of immune checkpoint blockade therapy *via* interferon-alpha response in muscle-invasive bladder cancer

**DOI:** 10.3389/pore.2023.1611117

**Published:** 2023-04-04

**Authors:** Bohan Fan, Xin Zheng, Yicun Wang, Xiaopeng Hu

**Affiliations:** ^1^ Department of Urology, Beijing Chao-Yang Hospital, Capital Medical University, Beijing, China; ^2^ Institute of Urology, Capital Medical University, Beijing, China; ^3^ Comprehensive Transplant Center, Northwestern University Feinberg School of Medicine, Chicago, IL, United States; ^4^ Department of Surgery, Northwestern University Feinberg School of Medicine, Chicago, IL, United States; ^5^ Department of Urology, Beijing Youan Hospital, Capital Medical University, Beijing, China

**Keywords:** muscle-invasive bladder cancer, immune checkpoint blockade therapy, interferon-alpha response, tumor microenvironment, prognostic biomarkers

## Abstract

**Background:** Immune checkpoint blockade (ICB) can prompt durable and robust responses in multiple cancers, involving muscle-invasive bladder cancer (MIBC). However, only a limited fraction of patients received clinical benefit. Clarifying the determinants of response and exploring corresponding predictive biomarkers is key to improving outcomes.

**Methods:** Four independent formerly published cohorts consisting of 641 MIBC patients were enrolled in this study. We first analyzed the associations between various cancer hallmarks and ICB therapy response in two immunotherapeutic cohorts to identify the leading prognostic hallmark in MIBC. Furthermore, advanced machine learning methods were performed to select robust and promising predictors from genes functioning in the above leading pathway. The predictive ability of selected genes was also validated in multiple MIBC cohorts.

**Results:** We identified and verified IFNα response as the leading cancer hallmark indicating better treatment responses, favorable overall survival, and an inflamed tumor microenvironment with higher infiltration of immune effector cells in MIBC patients treated with ICB therapy. Subsequently, two commonly selected genes, *CXCL10* and *LAMP3*, implied better therapy response and the CXCL10^high^LAMP3^high^ patients would benefit more from ICB therapy, which was comprehensively validated from the perspective of gene expression, clinical response, patient survival and immune features.

**Conclusion:** Higher IFNα response primarily predicted better ICB therapeutic responses and reflected an inflamed microenvironment in MIBC. A composite of *CXCL10* and *LAMP3* expression could serve as promising predictive biomarkers for ICB therapeutic responses and be beneficial for clinical decision-making in MIBC.

## Introduction

As one of the most lethal urinary malignancies worldwide, bladder cancer occurs with a high risk of treatment failure rate, recurrence and morbidity (1, 2). About 25% of patients would be initially diagnosed as muscle-invasive bladder cancer (MIBC) with a 5-year survival rate of less than 15% who did not receive intervention (3). Radical cystectomy complemented by cisplatin-based perioperative chemotherapy remains the mainstay of MIBC management. However, the treatment outcome and patient prognosis were still unsatisfying (4). Studies have recently shown that tumor immunotherapy, like immune checkpoint blockade (ICB), especially programmed cell death-1 (PD-1)/programmed cell death ligand-1 (PD-L1), could be used for PD-L1 immune positive and platinum ineligible patients, as well as newly for those who are responding to platinum as maintenance therapy, it could also invigorate antitumor immune response and prolong survival of advanced MIBC patients resistant to chemotherapy, which revolutionized the therapeutic landscape (5, 6). Nevertheless, as only a small subset of patients would benefit from ICB therapy, effective biomarkers were urgently required to predict patient responsiveness to ICB therapy (7).

Multiple biomarkers have been introduced to predict immunotherapeutic response, including PD-L1 expression level, tumor-specific neoantigens such as tumor mutational burden (TMB), and immune-infiltration indicative markers like gene-expression profile associated with T cell effector (3, 8). However, it was controversial to merely use PD-L1 as a biomarker considering its dynamic expression regulation (9). Besides, it has been well recognized that PD-L1 expression suggested a sustained immunosuppressive-factor-regulated immune response in the tumor microenvironment (10). Moreover, each of PD-L1, TMB and T cell-inflamed gene-expression profile could predict immunotherapy efficacy with only moderate correlation in previous studies (3, 11). Considering the economic burden, difficulties in detection and unsatisfying clinical needs, we attempt to correlate molecular mechanisms with clinical data to identify robust genes as potential biomarkers for therapy response prediction.

Interferon-α (IFNα), a cytokine belonging to type I IFN family can elicit robust immune responses and exert various antiviral and antitumor effects (12). IFNα enhances immune recognition by increasing class I and II MHC molecules expressions surfaced on tumor cells and it is regarded as a potential treatment strategy by directly blocking cell-cycle progression and promoting apoptosis, thus suppressing tumor extension through stimulating the expression of antitumor IFN-stimulated gene products and tumor suppressor proteins (13, 14). Moreover, IFNα plus PD-1 blockade was recently identified as a promising treatment strategy in melanoma and hepatocellular carcinoma (15–17). Despite such results, there was little understanding of the effects of IFNα response in ICB therapy of MIBC patients. In this research, IFNα response was first evaluated as the primary factor for the better prognosis of ICB therapy, then we employed advanced machine learning algorithms to further select eligible genes, which was validated in multiple immunotherapeutic MIBC cohorts.

## Materials and methods

### Data collection and preprocessing

Three independent cohorts consisting of MIBC patients treated with ICB therapy and the Cancer Genome Atlas (TCGA) cohort were included for analysis. The detailed patient characteristics can be seen in Table 1. Normalization and log_2_-transformation were conducted in all RNA-seq and microarray data.

**TABLE 1:** Clinical characteristics of MIBC patients in four independent cohorts.

Characteristics	IMvigor210	GSE176307	GSE111636	TCGA	Overall
Total	168	76	11	386	641
Application	Training	Validation I	Validation II	Validation III	
ICB Therapy (%)					
Atezolizumab	168 (100)	30 (39.5)			198 (77.6)
Pembrolizumab		40 (52.6)	11 (100)		51 (20.0)
Nivolumab		4 (5.3)			4 (1.6)
Avelumab		1 (1.3)			1 (0.4)
Durvalumab		1 (1.3)			1 (0.4)
Therapy response (%)					
CR	15 (8.9)	7 (9.2)	6 (54.5)	145 (37.6, *TIDE*)	207 (32.3)
PR	27 (16.1)	7 (9.2)			
SD	35 (20.8)	4 (5.3)	5 (45.5)	241 (62.4, *TIDE*)	434 (67.7)
PD	91 (54.2)	58 (76.3)			
Overall survival (%)					
Alive	61 (36.3)	28 (36.8)			89 (36.5)
Deceased	107 (63.7)	48 (63.2)			155 (63.5)
Follow-up time (Months, mean ± SD)	11.72 ± 7.48	7.97 ± 7.24			10.55 ± 7.59

The training cohort enrolled 168 MIBC patients three times weekly treated with 1,200 mg atezolizumab from IMvigor210 trial, patients with other cancer types or unconfirmed overall responses were excluded, corresponding data were downloaded by the IMvigor210CoreBiologies R package (18). A total of 76 MIBC patients treated with at least one dose of anti-PD-1 or anti-PD-L1 immunotherapy with response and survival information were recruited as validation I cohort (19). A small series of expression profiles taken from 11 MIBC patients treated with pembrolizumab was considered as validation II cohort used for expression validation. The last validation III cohort contained 386 MIBC patients from TCGA database. Full transcriptome data and characteristics of the patients were assessed from http://www.cbioportal.org/ in July 2022. Patients achieving complete response (CR) or partial response (PR) were regarded as responders, while patients with progressive disease (PD) or stable disease (SD) were defined as non-responders in the training and validation I cohort. Responses of immunotherapy in TCGA cohort were conducted using the Tumor immune dysfunction and exclusion (TIDE) analysis, responders were patients with TIDE score <0, otherwise, non-responders (20).

### Study design

**FIGURE 1:**
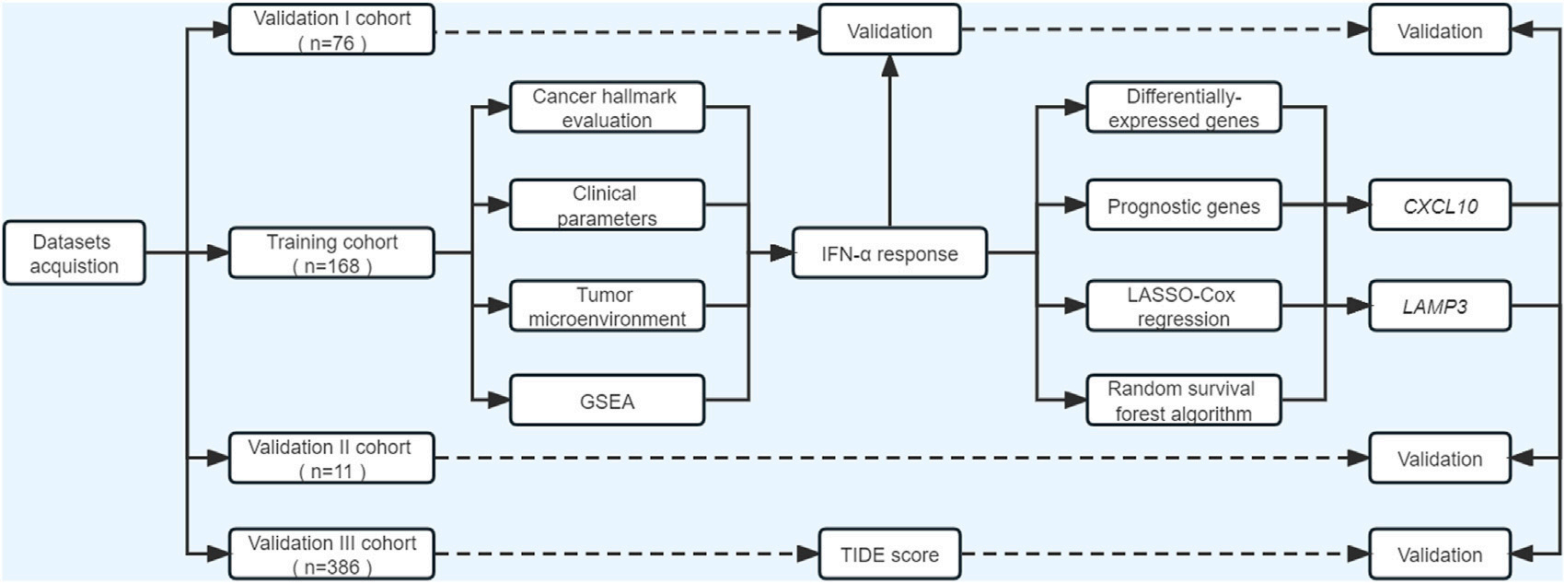
Flowchart of the current study. ssGSEA, single-sample gene set enrichment analysis; GSEA, gene set enrichment analysis; TIDE, Tumor immune dysfunction and exclusion.

As illustrated in Figure 1, our study included three phases. In the discovery phase, we measured the performances of 50 cancer hallmarks, identified and validated IFNα response as the leading factor for the favorable prognosis of ICB therapy from the survival, functional and tumor microenvironment perspectives. Secondly, differentially expressed prognostic genes mapping on IFNα response pathways were included for machine learning algorithms to select robust genes with better performance in therapy response prediction. Moreover, the expression levels and predictive abilities of the above candidate genes were verified in external validation cohorts.

### Cancer hallmark assessment

In the training cohort, we quantified levels of cancer hallmarks based on transcriptional profiles and hallmark gene sets acquired from the Molecular Signatures Database (MsigDB) through ssGSEA algorithms (21). Subsequently, the prognostic significance of cancer hallmarks was evaluated by univariate Cox analysis in MIBC patients after ICB therapy. We also applied gene set enrichment analysis (GSEA) to compare the enriched pathways between responders and non-responders referring to the hallmark gene sets (22). As a result, IFN-α response (IFNAR) was found with the lowest hazard ratio (HR) value. Subsequently, according to the IFNAR-related score, we divided patients into high-, middle- and low-score groups, the survival differences, responder proportion, PD-L1 protein expression levels on immune cells (IC) and immune phenotype were compared among groups.

### Tumor microenvironment evaluation

The overall infiltration of immune cells, stromal cells and tumor cell purity were inferred by ESTIMATE algorithm (23). Moreover, we evaluated the infiltration of 22 immune cell subpopulations in MIBC biopsies through CIBERSORT (Cell-type Identification by Estimating Relative Subsets of RNA Transcripts), a deconvolution algorithm to characterize immune cell composition using gene expression profiles (24). Immune cell abundance variations in the high- and low- IFNAR-related score groups were detected, correlations between immune cell infiltration and IFNAR were also calculated.

### Machine learning methods

Ninety-five genes that participated in the process of IFNAR were derived from MSigDB. We then screened differentially expressed genes (DEGs) between responders and non-responders through “limma” package when false discovery rate (FDR) was less than 0.05 (25). Meanwhile, prognostic genes were identified by univariate Cox analysis with a threshold of *p* < 0.05. Prognostic DEGs were selected for further analysis. Subsequently, two machine learning algorithms, least absolute shrinkage and selection operator (LASSO) Cox regression and random survival forest (RSF) analysis were commonly applied to perform gene selection. LASSO used 10-fold cross-validation to estimate the penalty parameters by “glmnet” package to avoid over-fitting. RSF adapts random forests to survival analysis based on ensemble trees. Variable importance (VIMP) evaluates the predictive ability alterations of RSF model when genes are randomly permuted, higher VIMP indicates greater significance, while the average depth of genes among all survival trees was implied by minimal depth, smaller values suggest increased importance (26). They measure the impact of genes from different points of view, eligible genes identically selected by LASSO, VIMP and minimal depth were obtained for further validation.

### Tumor immune dysfunction and exclusion analysis

To predict the therapeutic response to ICB therapy of MIBC patients in TCGA cohort, we applied TIDE algorithm to evaluate diverse mechanisms in tumor immune evasion, comprising of immunosuppressive factors induced cytotoxic T lymphocytes (CTLs) exclusion and dysfunction. Before analysis in TCGA cohort, we conducted TIDE in the training and validation I cohort to test its predictive ability in ICB therapy response. The infiltration of immunosuppressive myeloid suppressor cells (MDSC), cancer-associated fibroblasts (CAFs) and M2 subtypes of tumor-associated macrophages (TAM.M2) were also evaluated (20). Lower TIDE scores imply better clinical efficacy of immune checkpoint inhibitors.

### Statistical analysis

We performed all statistical analyses with R software. The D’Agostino and Pearson omnibus normality tests were initially carried out to assess whether the data fit a normal distribution. When parameters were normally distributed, a two-tailed unpaired t-test, one-way ANOVA with Tukey’s correction and the Pearson correlation would be conducted. Once data did not achieve the assumptions of parametric tests, the Mann–Whitney U test, one-way ANOVA using Kruskal–Wallis with Dunn’s correction and Spearman correlation would be employed. Results met the level of 5% (*p* < 0.05) were considered statistically significant.

## Results

### Identification and validation of IFNα response as the leading favorable factor for the prognosis of ICB therapy

**FIGURE 2:**
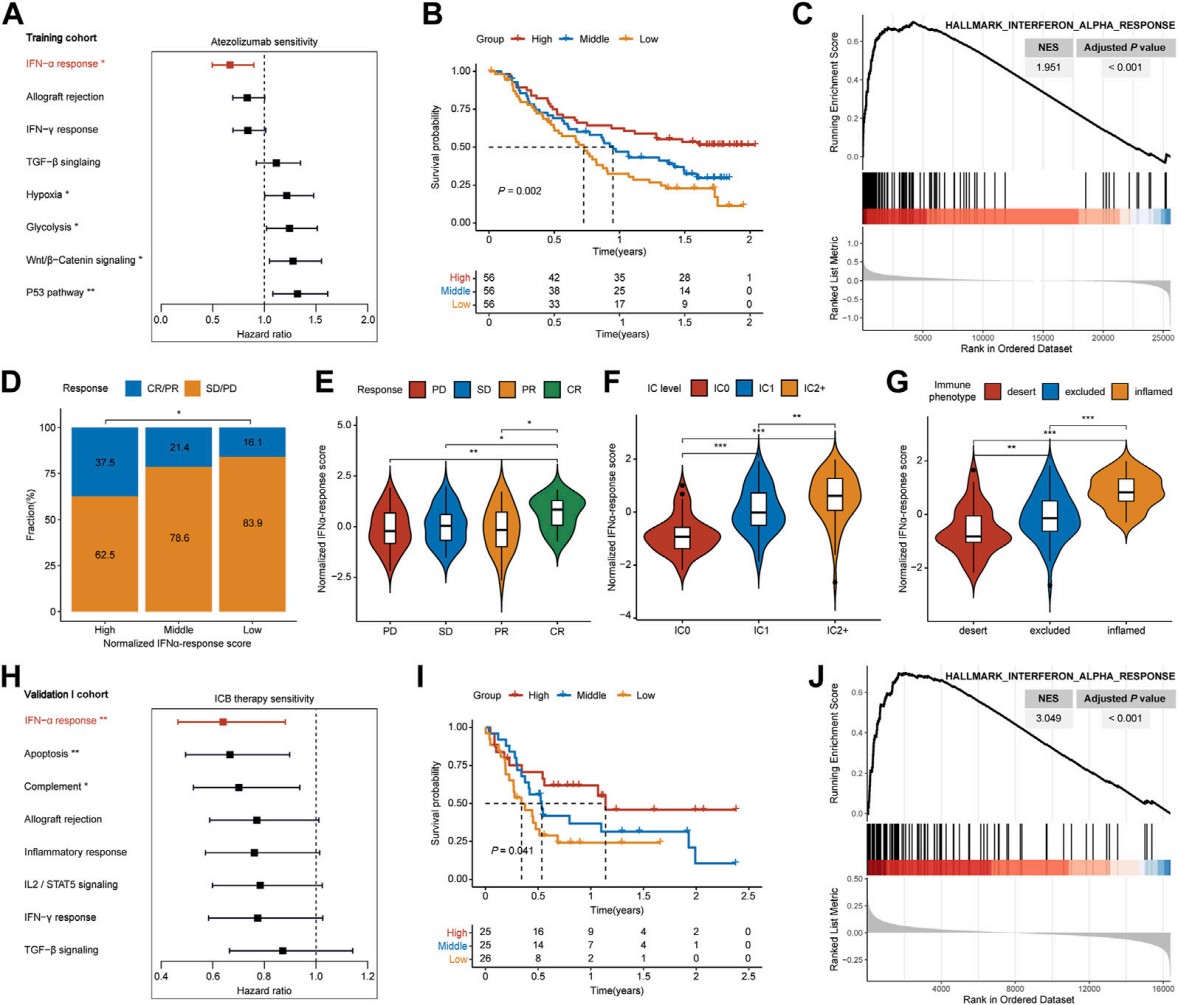
Detection of IFNα response as the leading favorable factor for therapeutic response and prognosis of ICB therapy in MIBC. In the training cohort, **(A)** forest plot shows that IFNα response has the lowest hazard ratio with statistical significance among various cancer hallmarks, **(B)** Kaplan–Meier survival curves depict that higher IFNAR-related score was associated with better overall survival, **(C)** GSEA plot illustrates that IFNα response is significantly enriched in responders than non-responders, **(D)** higher IFNAR-related score present significantly higher percentages of responses (CR/PR) and lower percentages of non-responses (SD/PD), **(E–G)** violin plot show that the IFNAR-related score was higher in patients with CR, the higher protein expression level of PD-L1 on IC, and inflamed immune phenotype. In the validation I cohort, **(H,I)** IFNα response was similarly regarded as a positive indicator for patient prognosis, **(J)** and it was significantly higher enriched in responders. NES, normalized enrichment score; CR, complete response; PR, partial response; SD, stable disease; PD, progressive disease; IC, immune cells. ∗*p* < 0.05, ∗∗*p* < 0.01, ∗∗∗*p* < 0.001.

In the training cohort, we quantified the performance of 50 cancer hallmarks, then each HR value was calculated and ranked through univariate Cox analysis (Supplementary Table S1; Supplementary Figure S1). Among hallmarks, IFNAR ranked first with the lowest HR value (Figure 2A, HR = 0.667, *p* = 0.008), and high IFNAR-related score indicated favorable overall survival (OS) (Figure 2B, *p* = 0.002). Moreover, GSEA also revealed that IFNAR was significantly higher enriched in patients with responses to ICB therapy (Figure 2C; Supplementary Table S2). Based on the IFNAR-related score, we equally divided patients into three groups, the fraction of patients achieving CR or PR were 37.5%, 21.4%, and 16.1% in the high-, middle- and low-score group, respectively (Figure 2D, *p* = 0.024). Besides, the IFNAR-related score was higher in the group of patients achieving CR, patients with the higher protein expression level of PD-L1 on IC and patients with inflamed immune phenotype than other groups (Figures 2E–G). Likewise, in the validation I cohort, IFNAR was ordered first among cancer hallmarks (Figure 2H, HR = 0.640, *p* = 0.006) (Supplementary Table S3), high IFNAR-related score was linked to better OS (Figure 2I, *p* = 0.041), IFNAR was significantly upregulated in responders (Figure 2J; Supplementary Table S4). Together, our study suggested that the IFNAR pathway was a leading favorable factor with promising predictive value for the prognosis of ICB therapy in MIBC patients.

### IFNα response ignites inflamed tumor microenvironment in MIBC

**FIGURE 3:**
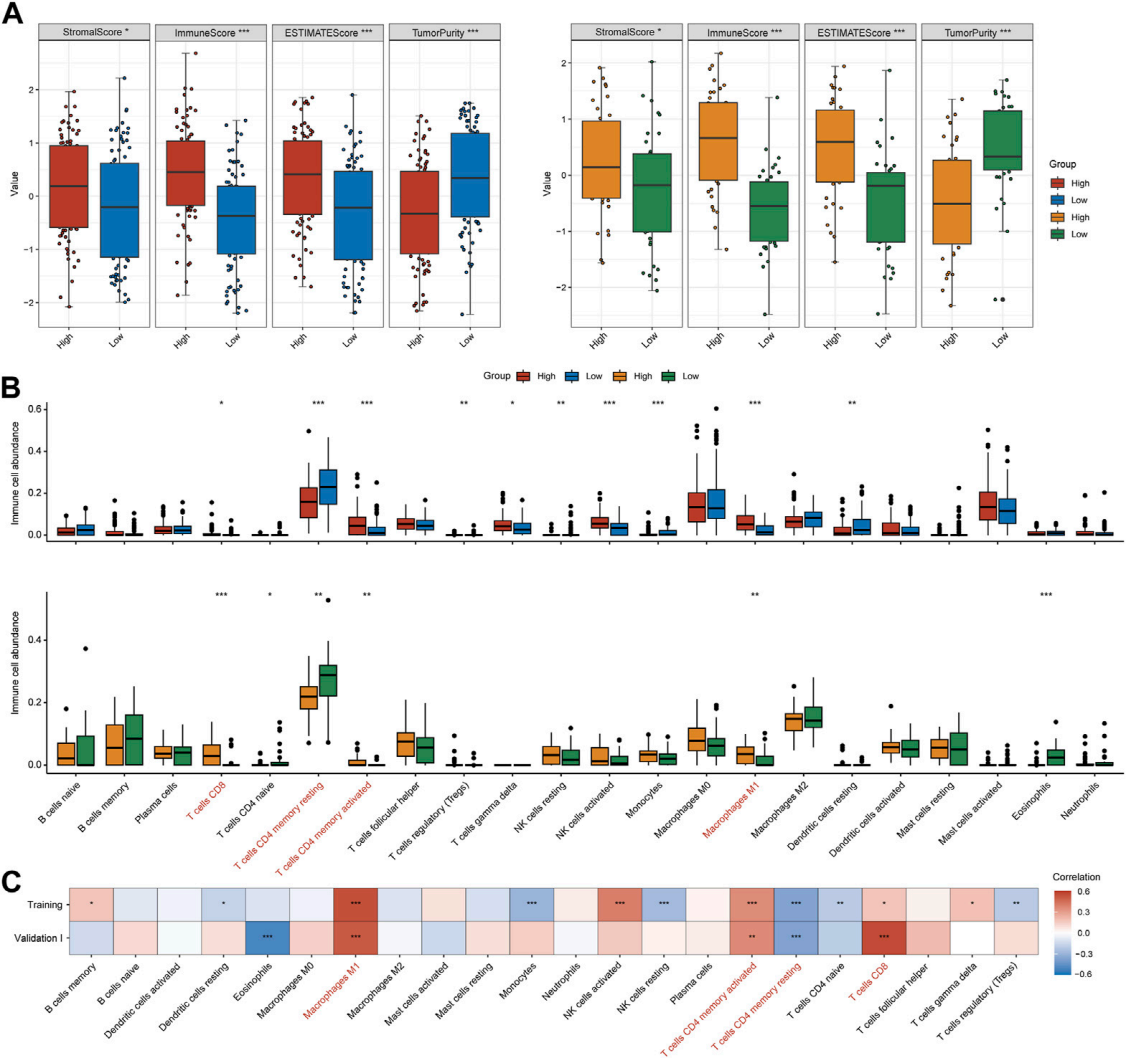
IFNα response represents an inflamed immune context in MIBC. In the training and validation I cohort, **(A)** box plots display that higher immune score, stromal score and lower tumor purity were seen in the high-IFNAR-related-score subgroup, **(B)** several types of immune effector cells including CD4^+^ memory-activated T cells, CD8^+^ cells and macrophage M1 were higher infiltrated, while CD4^+^ memory-resting T cells were lower infiltrated in the tumor microenvironment of MIBC patients with high IFNAR-related score. **(C)** The correlation heatmap shows the association between IFNAR-related score and immune cell infiltration. The blanks are filled in proportion to Spearman’s coefficient values, positive and negative correlations are colored in red and blue, respectively. ∗*p* < 0.05, ∗∗*p* < 0.01, ∗∗∗*p* < 0.001.

It has been well established that the tumor microenvironment influences clinical efficacy of immunotherapy. Therefore, we utilized ESTIMATE and CIBEROSRT to calculate the immune cell infiltration in tumor tissues. Both for training and validation I cohorts, the high-IFNAR-related-score subgroup exhibited higher immune and stromal scores, lower tumor purity than the low-score subgroup did (Figure 3A). In particular, immune effector cells, including CD8^+^ T cells, CD4^+^ memory-activated T cells and type 1 proinflammatory macrophage (M1) were increasingly infiltrated, while CD4^+^ memory-resting T cells were decreasingly infiltrated in the high-IFNAR-related-score subgroup in both cohorts (Figure 3B). Furthermore, the IFNAR-related score was positively correlated with above mentioned immune effector cells and negatively associated with CD4^+^ memory-resting T cells (Figure 3C). Collectively, these results illustrated high levels of IFNAR were accompanied by an immune-active tumor microenvironment in MIBC.

### Detection of *CXCL10* and *LAMP3* for the prediction of ICB therapy response

**FIGURE 4:**
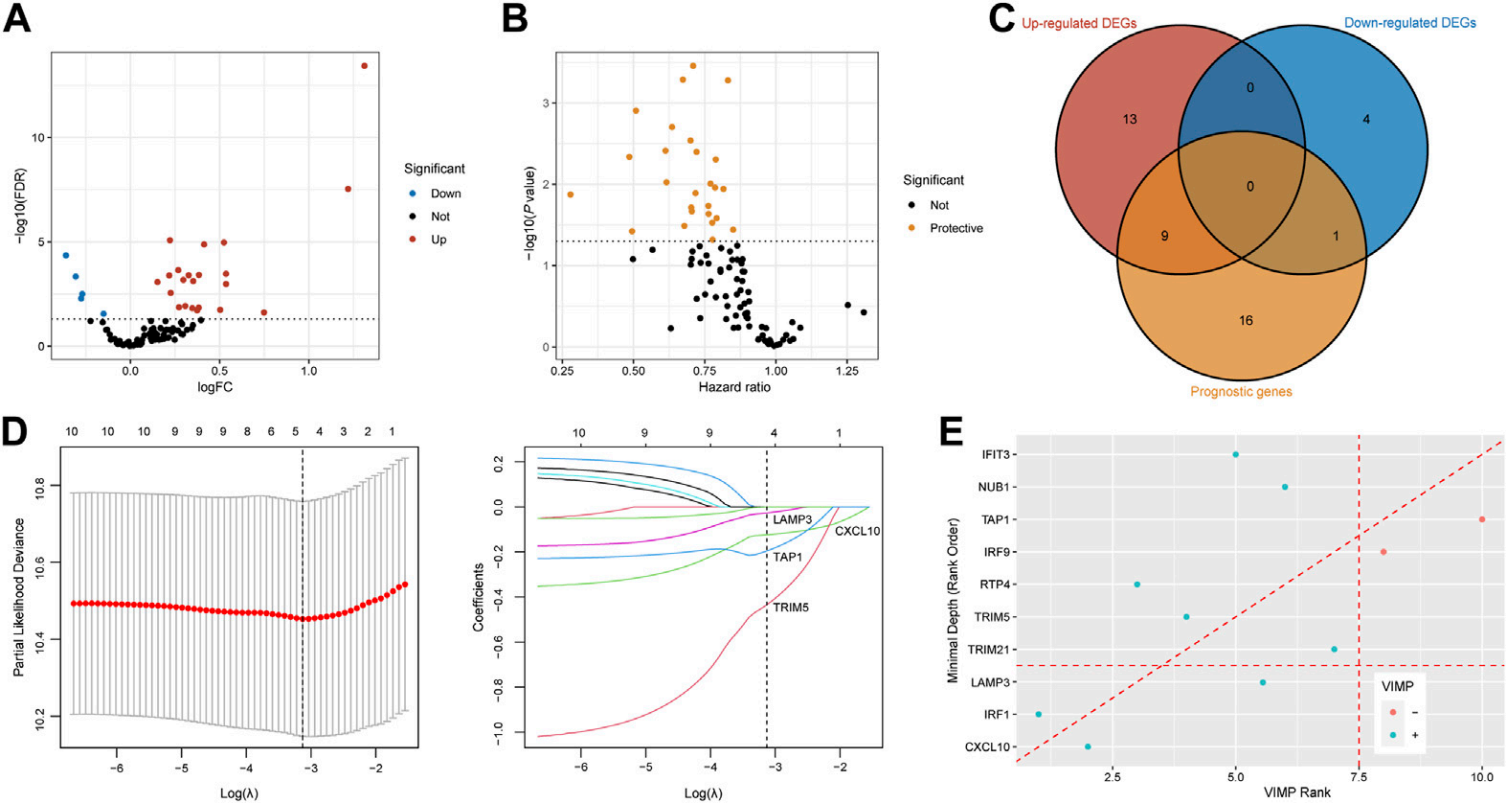
Recognition of *CXCL10* and *LAMP3* for immunotherapeutic response prediction by machine learning methods. **(A,B)** Volcano plots show DEGs between responders and non-responders and prognostic genes calculated by the univariable Cox regression. Red dots are upregulated genes, blue dots present downregulated genes, protective genes are dotted in yellow. **(C)** Venn diagram shows 10 intersected genes between DEGs and prognostic genes. **(D)** Tenfold cross-validation was utilized to calculate optimal lambda which leads to minimum mean cross-validation error by LASSO Cox regression analysis. Four genes were finally selected under the optimal lambda. **(E)** Variable importance plot of the random survival forest analysis comparing rankings with VIMP and minimal depth. The VIMP rank is reported on the x-axis and minimal depth (rank order) is on the y-axis. The horizontal line indicates the minimal depth threshold, important variables are below the line. The vertical line divides variables with positive VIMP (left) from those with negative VIMP (right, unimportant). *CXCL10* and *LAMP3* were commonly selected by LASSO, VIMP and minimal depth. FDR, false discovery rate; FC, fold change; DEGs, differentially expressed genes; VIMP, variable importance.

We acquired IFNAR-related genes (*n* = 95) for subsequent analyses. With the threshold of FDR <0.05, twenty-four upregulated DEGs and 5 downregulated DEGs in tumor tissues of responders compared to non-responders were identified (Figure 4A). Meanwhile, seventeen prognostic genes with *p* < 0.05 were also discovered (Figure 4B). Ten intersected prognostic DEGs were then applied to machine learning algorithms (Figure 4C). Then four genes (*CXCL10*, *LAMP3*, *TAP1*, *TRIM5*) were selected by LASSO Cox regression analysis (Figure 4D), three genes (*CXCL10*, *LAMP3*, *IRF1*) were acquired based on VIMP and minimal depth through RSF (Figure 4E). Among them, *CXCL10* and *LAMP3* were co-selected as promising predictive biomarkers for the responses of ICB therapy.

### *CXCL10* and *LAMP3* show robust predictive ability in the clinical benefits of ICB therapy

**FIGURE 5:**
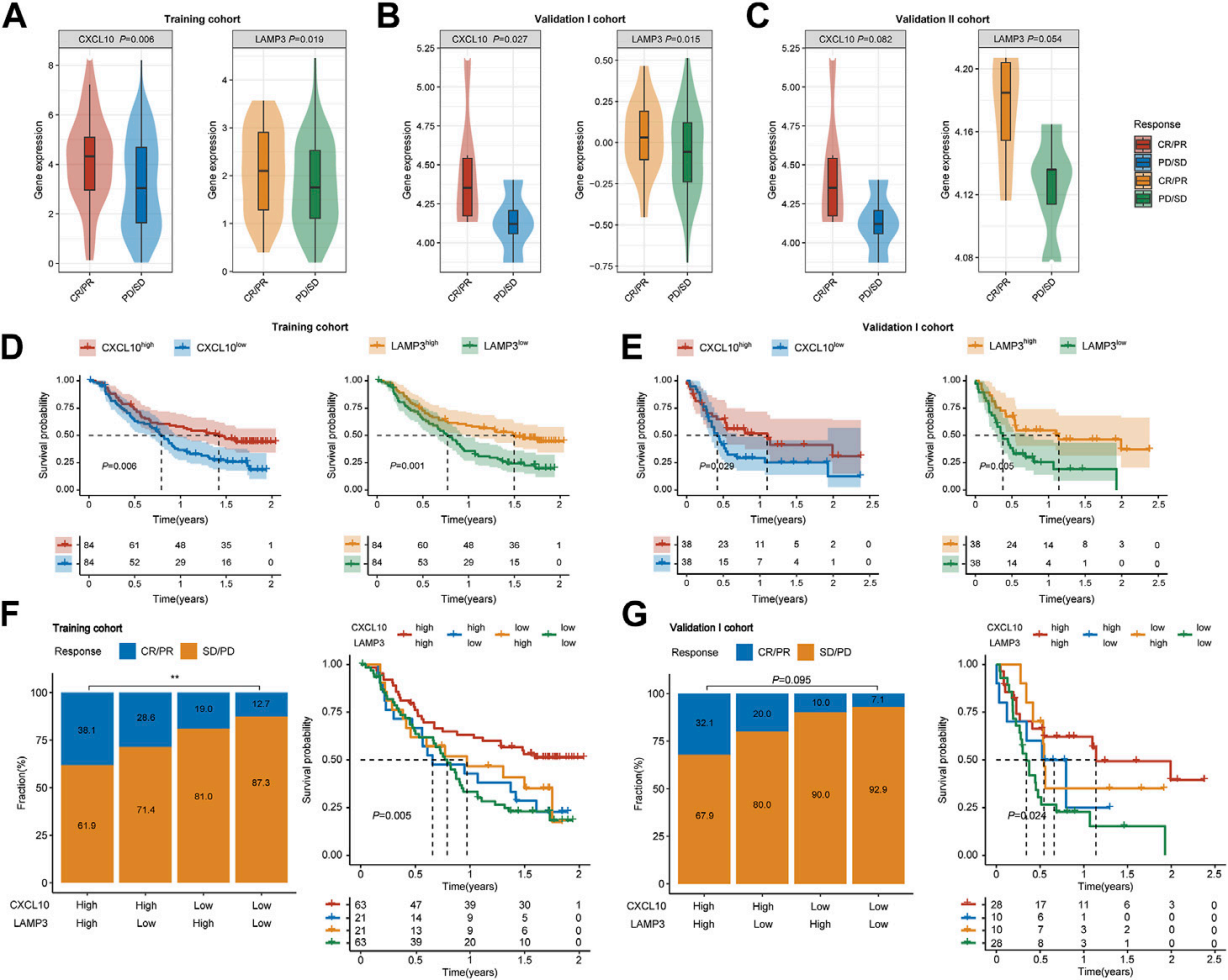
Expression and predictive ability validation of *CXCL10* and *LAMP3* in multiple cohorts. **(A–C)** Variations of *CXCL10* and *LAMP3* mRNA expression between the responder and non-responder group in the training and validation I and II cohorts. **(D,E)** Kaplan-Meier analyses of overall survival in patients in the training and validation I cohort, stratified according to the median values of *CXCL10* and *LAMP3* mRNA expression. Data were analyzed by log-rank test. **(F,G)** Clinical response to ICB therapy and Kaplan-Meier analyses of overall survival stratified according to the combination of *CXCL10* and *LAMP3* in the training and validation I cohort. ∗∗*p* < 0.01.

In the training cohort, *CXCL10* and *LAMP3* were significantly higher expressed in responders (Figure 5A, *p* = 0.006, *p* = 0.019, respectively). The same trend was also observed in another two validation cohorts (Figures 5B, C). Besides, both *CXCL10* and *LAMP3* expression in the tumor tissues indicated favorable OS in the training cohort (Figure 5D, *p* = 0.006, *p* = 0.001, respectively) and validation I cohort (Figure 5E, *p* = 0.029, *p* = 0.005, respectively). Moreover, we noted the patients who achieved CR or PR were more frequent in *CXCL10* ^*high*^ *LAMP3* ^*high*^ subgroups, and the combination of *CXCL10* and *LAMP3* expression significantly indicated improved OS in both cohorts (Figures 5F, G). Moreover, the expression levels of *CXCL10* and *LAMP3* were higher in patients with inflamed immune phenotype and higher PD-L1 protein expression levels on IC than in other groups (Supplementary Figure S2). Together, our study suggested that *CXCL10* plus *LAMP3* could serve as an ideal and stable predictive biomarker for patient response to ICB therapy in clinical settings.

### Higher IFNα response, expression levels of *CXCL10* and *LAMP3* indicate better ICB therapy response in TCGA cohort

**FIGURE 6:**
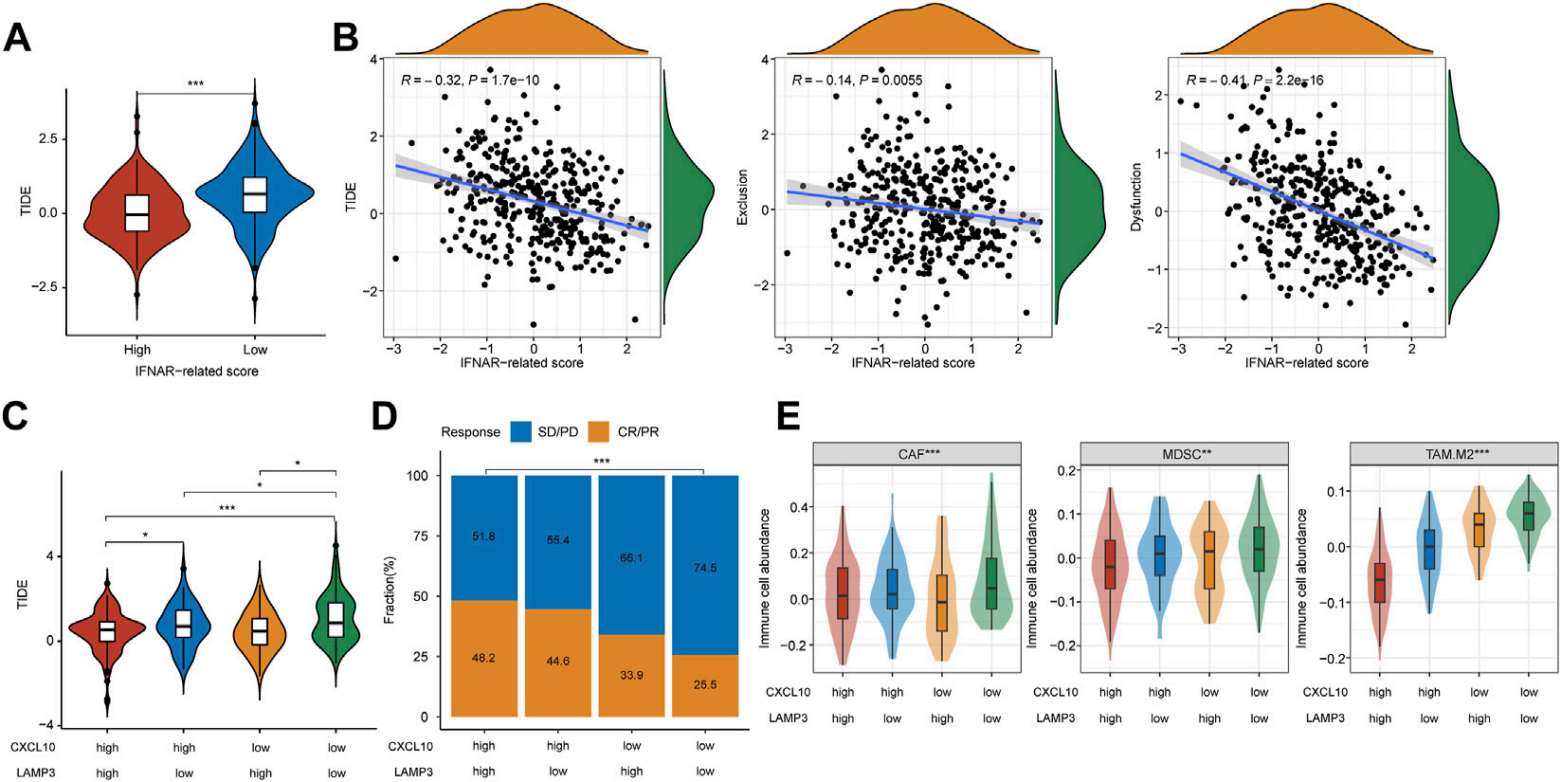
*CXCL10* and *LAMP3* expression indicate estimated ICB therapy benefit in TCGA cohort. **(A)** TIDE score was lower in the high-IFNAR-related-score subgroup, indicating better ICB therapy response. **(B)** Correlation analysis between IFNAR-related score and TIDE, tumor immune exclusion and dysfunction scores. **(C)** TIDE score was higher in the CXCL10^low^LAMP3^low^ subgroup, indicating worse ICB therapy response. **(D,E)** Differences in clinical response and three types of immunosuppressive cell infiltration including tumor-associated fibroblast (CAF), myeloid-derived suppressor cell (MDSCs) and M2 subtype of tumor-associated macrophage (TAM.M2) among groups. ∗*p* < 0.05, ∗∗*p* < 0.01, ∗∗∗*p* < 0.001.

In the training and validation I cohort, lower TIDE scores were seen in patients with CR/PR, and patients in the high-TIDE-score group showed worse overall survival (Supplementary Figure S3), which proved the predictive ability of TIDE in ICB therapy response. To further evaluate the universal applicability of *CXCL10* and *LAMP3* in predicting the responsiveness to ICB therapy, we evaluated therapy responses of MIBC patients through TIDE algorithm in TCGA cohort. The TIDE score was significantly higher in the low-IFNAR-related-score subgroup, which indicates lower therapeutic sensitivity (Figure 6A, *p < 0.001*). Furthermore, a strong correlation was found between IFNAR-related scores and TIDE scores (*R* = −0.32, *p* = 1.7e-10), the IFNAR-related score was also negatively associated with exclusion (*R* = −0.14, *p* = 0.006) and dysfunction (*R* = −0.41, *p* = 2.2e–16) (Figure 6B). As for the combination of *CXCL10* and *LAMP3*, the TIDE score was lower and the responder frequency was higher in the *CXCL10* ^*high*^ *LAMP3* ^*high*^ subgroup (Figures 6C, D). Intriguingly, the scores of three reported immunosuppressive cells that suppress tumor T-cell infiltration, consisting of CAFs, MDSCs and TAM.M2 were higher in the *CXCL10* ^*low*^ *LAMP3* ^*low*^ subgroup (Figure 6E). The above results implied that high expression levels of *CXCL10* and *LAMP3* may predict a tumor microenvironment that favors immunotherapeutic response and indicate better ICB therapy response.

## Discussion

ICB therapy has revolutionized cancer management in the last few years, and it could improve the prognosis of platinum-refractory advanced MIBC patients (6, 27). Despite considerable progress, immune checkpoint inhibitors could benefit only a subset of patients, with the incidence of adverse events up to 16% (28). Elucidation of the underlying characteristics will better identify patients who will be more benefited from ICB therapy.

IFNα, collectively known as type I IFNs, functions as a dynamic immune mediator that orchestrates both innate and adaptive antitumor immune responses (29). Although IFNα is not individually used for cancer treatment anymore due to its systematic side effects, its large impacts on the immune system hold immense potential for IFNα to elicit a cytotoxic immune response thus serving as a promising adjuvant agent with PD-1/PD-L1 inhibitors (16, 30). Recent clinical trials and preclinical models proposed that IFNα plus an anti-PD-1 antibody was an efficient treatment strategy in cancer, emphasizing the great potential of IFNα-based combination ICB (15, 31, 32). In this research, we first identified and validated IFNα response as a dominant favorable factor for the therapy response and prognosis of ICB therapy in two independent cohorts. Patients with higher IFNAR-related scores were associated with better therapeutic response and ignites inflamed immune context in MIBC, our results further demonstrated its great potential in improving and predicting ICB therapy response. In the previous IMvigor210 study, TGFβ signaling was found to attenuate tumor response to PD-L1 blockade. However, no clear difference was seen in the overall survival of patients with different TGFβ signaling scores in our study, possibly because of the inclusion of only bladder cancer patients, while the IMvigor210 study included other cancer types.

Due to powerful immunostimulatory properties, CD4^+^ T cells have been recognized to play essential roles in augmenting endogenous immune response (33). A recent study demonstrated that the predominance and persistence of CD4^+^ T cells could induce decade-long leukemia remission (34). Therefore, stimulating CD4^+^ T cells is crucial to achieving long-term antitumor immune memory in cancer immunotherapy (35). In this research, CD4^+^ memory T cells take up high contents in the tumor environment. Besides, the IFNAR-related score was positively corrected with CD4^+^ memory-activated T cells and negatively associated with CD4^+^ memory-resting T cells. It has also been reported that an increased amount of CD4^+^ effector memory T cells was found in IFNα treated chronic myeloid leukemia patients (36). Apart from this, the repolarization of macrophages from a pro-tumor phenotype (M2) to cytotoxic anti-tumor effectors (M1) is expected to refine the tumor environment and promote anti-tumor response (37). Similarly, higher infiltration of macrophage M1 was also seen in the high IFNAR-related score group. Collectively, our results illustrated that high IFNα response represents an inflamed immune microenvironment and further confirmed its promising role in predicting the therapeutic responses of ICB therapy in MIBC.

Migration and trafficking of CD8^+^ effector T cells into the tumor microenvironment along with sensing of chemokine gradients are essential to immunotherapy efficacy (38, 39), which is consistent with our results that more CD8^+^ T cells were infiltrated in the high-IFNAR-related-score subgroup. Preclinical studies have illustrated that chemokines C-X-C motif chemokine ligand 9 (CXCL9) and CXCL10 predominantly drive the recruitment of activated CD8^+^ T cells into tumor sites by engaging the corresponding chemokine receptor CXCR3 expressed on immune cells, with CXCL10 being more abundantly expressed (38, 40, 41). Therefore, strategies *via* induction of CXCL10 to support effector T cell recruitment have been considered as a mechanism-based intervention to enhance immunotherapy efficacy (10). Furthermore, CXCL10 expression in tumor tissues has been reported to be strongly associated with responses to ICB therapy (38, 42), which is consistent with our results. LAMP3 (lysosome-associated membrane protein 3), a dendritic cell (DC)—specific glycoprotein induced upon DC maturation after inflammatory stimulation that leads to primary T-cell responses (43). In patients with IIIA non-small cell lung cancer after neoadjuvant pembrolizumab and chemotherapy, LAMP3+ DCs involved in the process of lymphocytes recruitment and regulation, its increased levels were found to be associated with positive clinical outcomes by single-cell profiling (44). Besides, LAMP3 was also reported to be in the immunotherapy-response-associated signature of tertiary lymphoid structures in melanoma (45). As a consequence, the predictive ability of *CXCL10* and *LAMP3* expression was sensible, illustrating the interplay between immunity and cancer, which could better reflect the therapeutic responsiveness in MIBC patients.

Several limitations existed in this study. We included an ICB untreated TCGA dataset as the validation III cohort, and the TIDE score was applied as the surrogate endpoint, which may not align with actual therapy responses. Moreover, the training cohort included only atezolizumab-treated patients while the validation cohorts were either mixed or included only pembrolizumab-treated patients, which may be confounding factors. This study was a retrospective analysis including four independent cohorts to assess the ability of IFNα response, the combined *CXCL10* and *LAMP3* expression in predicting the clinical efficiency of ICB therapy, which needs to be verified in a larger and prospective trial in the future. Due to the lack of complete clinicopathological information, we should also correlate our results with clinical characteristics in further study. Besides, immune cell abundance and functional enrichment analysis were estimated by bioinformatic approaches in this study, lacking direct evidence and requiring further experimental verification.

## Conclusion

In summary, we identified IFNα response as the primary indicator associated with better ICB therapeutic response and an immune-inflamed microenvironment, and the combination of *CXCL10* and *LAMP3* expression could serve as effective predictive biomarkers for ICB treatment response and would be beneficial for patient-tailored treatment decisions in MIBC.

## Data Availability

Corresponding author may be contacted for article data if there is a valid reason.

## Supplementary material

SUPPLEMENTARY FIGURE S1 Higher TGFβ-signaling-score showed no survival differences in both training and validation I cohorts, but indicated worse overall survival in the IMvigor210 whole cohort.

SUPPLEMENTARY FIGURE S2 Patients with higher IC levels or inflamed immune phenotype had higher expression levels of *CXCL10* and *LAMP3*..

SUPPLEMENTARY FIGURE S3 Higher TIDE score was associated with better ICB therapy responses and overall survival in both training and validation I cohort.

SUPPLEMENTARY TABLE S1 Univariate Cox analysis of cancer hallmarks in the training cohort.

SUPPLEMENTARY TABLE S2 Gene set enrichment analysis in the training cohort.

SUPPLEMENTARY TABLE S3 Univariate Cox analysis of cancer hallmarks in the validation I cohort.

SUPPLEMENTARY TABLE S4 Gene set enrichment analysis in the validation I cohort.
