# Supplementary figures and images for: Predicting prognosis and clinical efficacy of immune checkpoint blockade therapy via interferon-alpha response in muscle-invasive bladder cancer
Source: Pathol Oncol Res. 2023 Apr 4;29:1611117. doi: 10.3389/pore.2023.1611117 (PMC10110843; doi:10.3389/pore.2023.1611117)

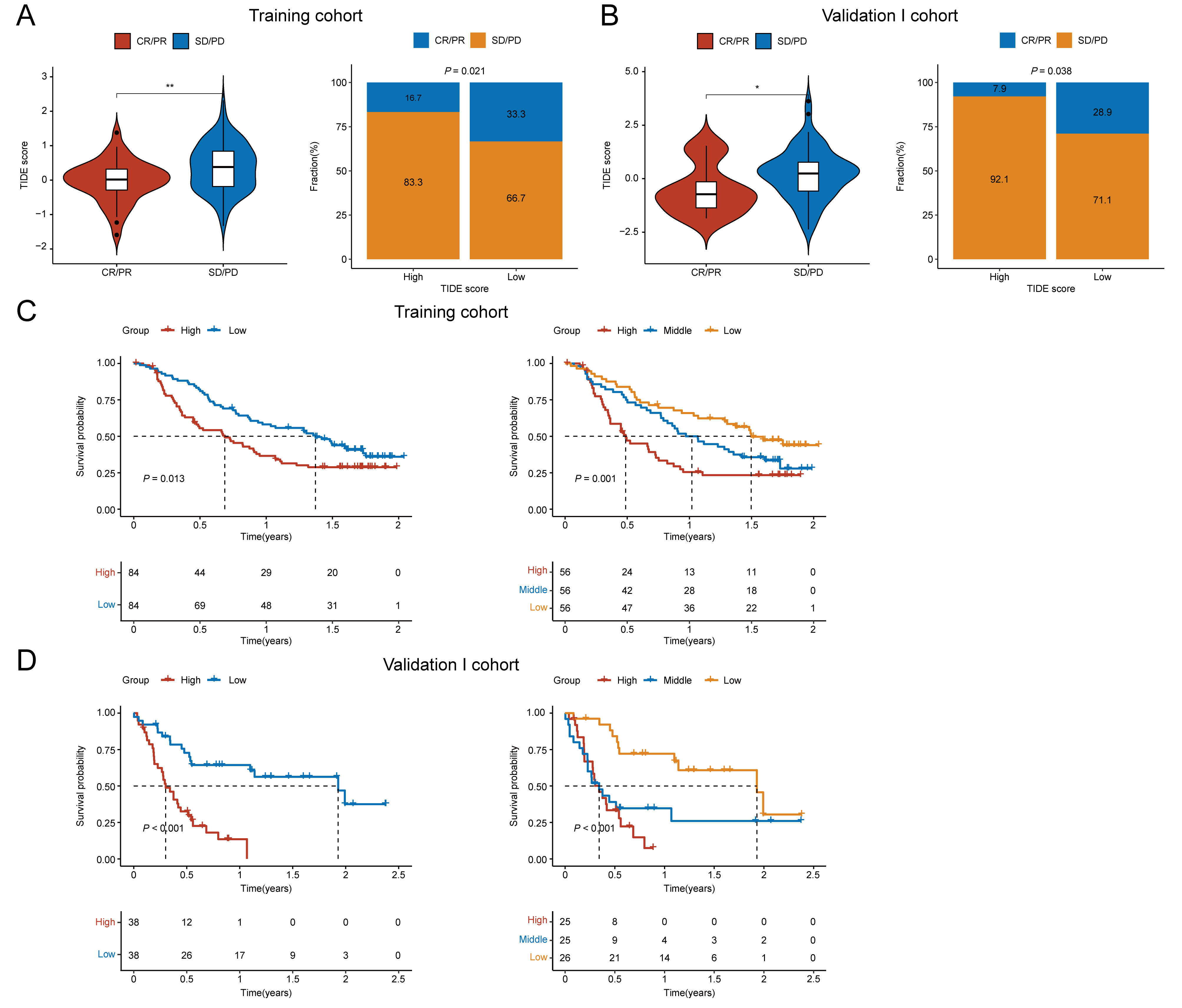

Supplement: Supplementary file 1 [file Image3.TIF]

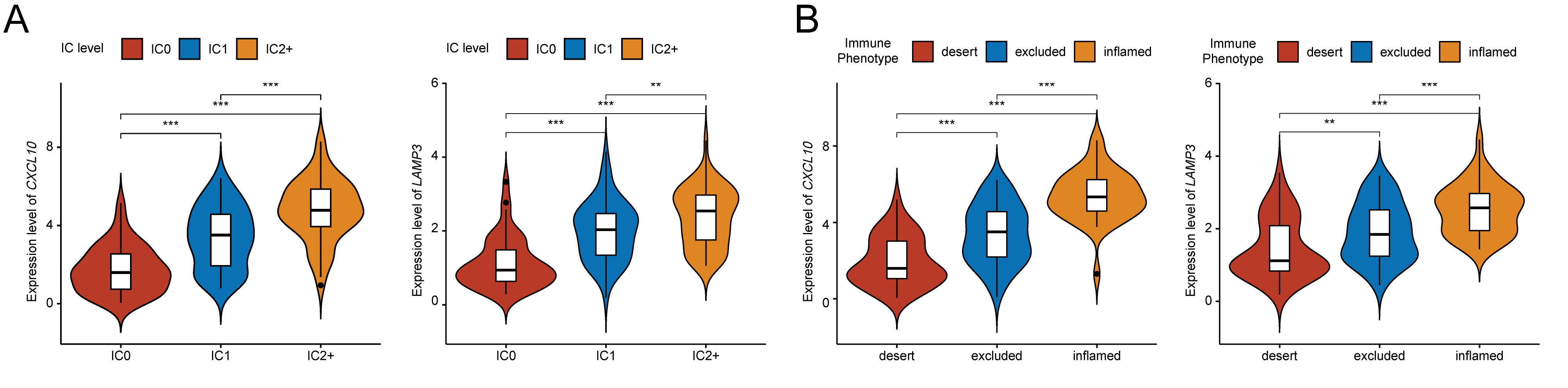

Supplement: Supplementary file 2 [file Image2.TIF]

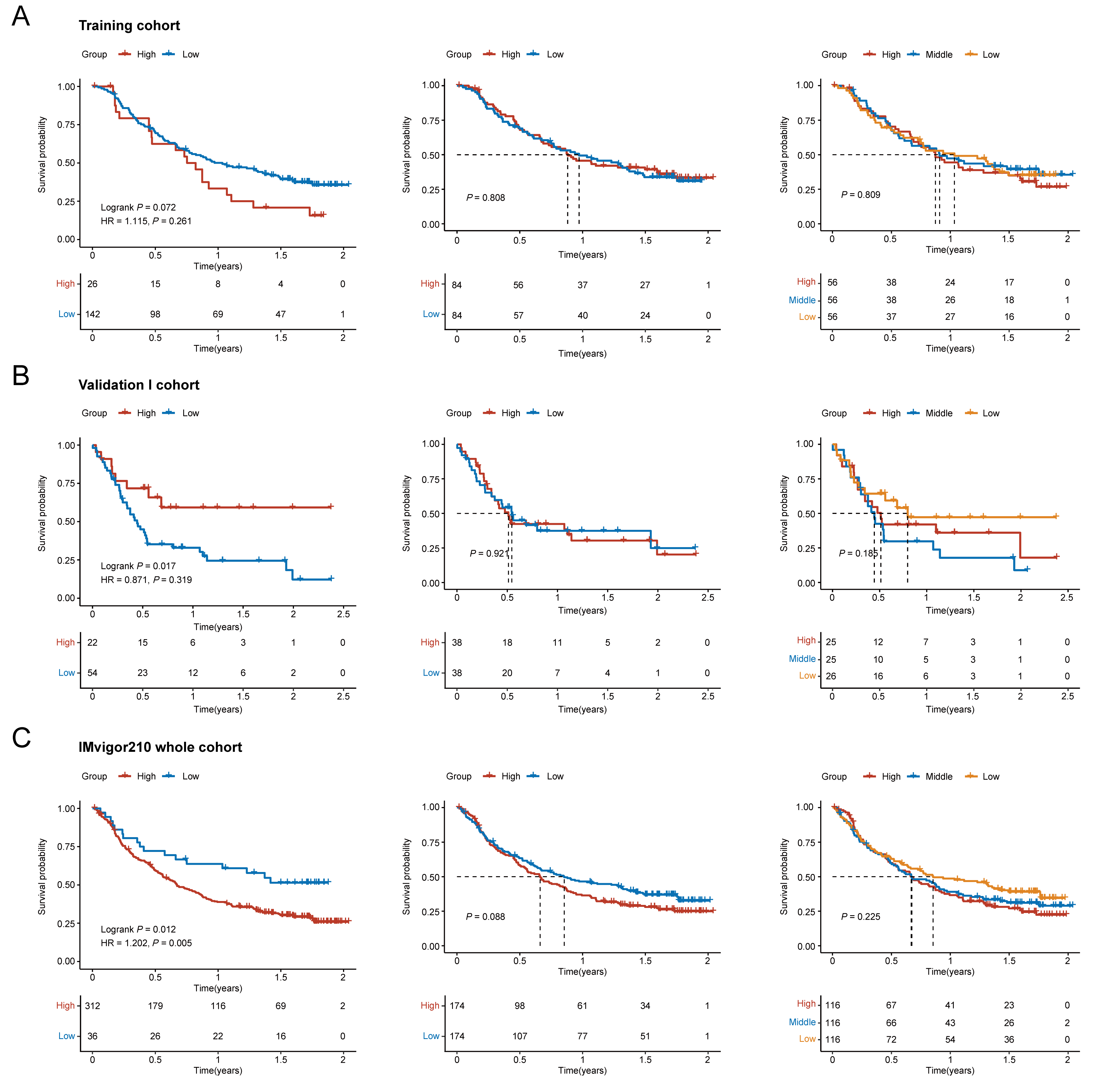

Supplement: Supplementary file 3 [file Image1.TIF]
