# Supplementary material for: Predicting prognosis and clinical efficacy of immune checkpoint blockade therapy via interferon-alpha response in muscle-invasive bladder cancer
Source: Pathol Oncol Res. 2023 Apr 4;29:1611117. doi: 10.3389/pore.2023.1611117 (PMC10110843; doi:10.3389/pore.2023.1611117)
Supplement: Supplementary file 4 [file DataSheet1.docx]

Table S1: Univariate Cox analysis of 50 cancer hallmarks in the training cohort.

| ID | HR | HR.95L | HR.95H | *P* value |
| --- | --- | --- | --- | --- |
| P53 pathway | 1.320899 | 1.081681 | 1.613022 | 0.00633 |
| IFN-α response | 0.666838 | 0.49531 | 0.897766 | 0.007567 |
| Wnt/β-Catenin signaling | 1.276557 | 1.049157 | 1.553243 | 0.014712 |
| Glycolysis | 1.242573 | 1.021092 | 1.512094 | 0.030132 |
| Hypoxia | 1.217001 | 1.002838 | 1.4769 | 0.046739 |
| IFN-γ response | 0.834089 | 0.693953 | 1.002523 | 0.053223 |
| Allograft rejection | 0.839725 | 0.696393 | 1.012558 | 0.067358 |
| Late estrogen response | 1.201665 | 0.977455 | 1.477304 | 0.081239 |
| Early estrogen response | 1.182155 | 0.965714 | 1.447105 | 0.104838 |
| Notch signaling | 1.165077 | 0.95702 | 1.418365 | 0.127942 |
| Spermatogenesis | 0.875223 | 0.72942 | 1.05017 | 0.151727 |
| Reactive oxygen species pathway | 1.14415 | 0.934867 | 1.400286 | 0.191378 |
| Down-regulated in KRAS signaling | 1.128931 | 0.933678 | 1.365016 | 0.210687 |
| MYC targets v2 | 1.131484 | 0.929219 | 1.377776 | 0.218932 |
| Apical junction | 1.127114 | 0.925138 | 1.373186 | 0.234968 |
| PI3K/AKT/mTOR signaling | 0.89378 | 0.737514 | 1.083156 | 0.252089 |
| Pancreatic β cells | 0.893688 | 0.735766 | 1.085504 | 0.257229 |
| TGF-β signaling | 1.11543 | 0.92211 | 1.34928 | 0.260624 |
| IL6/JAK/STAT3 signaling | 0.900896 | 0.74816 | 1.084813 | 0.270859 |
| E2F targets | 0.900874 | 0.747545 | 1.085652 | 0.272799 |
| Protein secretion | 0.918286 | 0.755516 | 1.116123 | 0.391806 |
| Oxidative phosphorylation | 1.094218 | 0.88386 | 1.354641 | 0.408465 |
| Heme metabolism | 1.07731 | 0.897096 | 1.293726 | 0.425278 |
| G2M checkpoint | 0.928826 | 0.770476 | 1.119719 | 0.438796 |
| Adipogenesis | 1.073538 | 0.879532 | 1.310336 | 0.485337 |
| Complement | 0.934615 | 0.772457 | 1.130814 | 0.486737 |
| IL2/STAT5 signaling | 0.935933 | 0.775087 | 1.130158 | 0.491332 |
| Angiogenesis | 1.064235 | 0.876903 | 1.291586 | 0.528551 |
| TNF-α signaling via NF-κB | 1.058558 | 0.880527 | 1.272584 | 0.54471 |
| Hedgehog signaling | 1.05948 | 0.875302 | 1.282412 | 0.553176 |
| Androgen response | 0.947864 | 0.787159 | 1.141378 | 0.572151 |
| Mitotic spindle | 0.950444 | 0.789014 | 1.144903 | 0.592542 |
| Fatty acid metabolism | 1.052187 | 0.853136 | 1.29768 | 0.634469 |
| Epithelial-mesenchymal transition | 1.044331 | 0.860552 | 1.267359 | 0.660495 |
| MYC targets v1 | 1.044523 | 0.854087 | 1.27742 | 0.671447 |
| Inflammatory response | 0.960393 | 0.795716 | 1.159151 | 0.673687 |
| Coagulation | 1.040749 | 0.852455 | 1.270635 | 0.694878 |
| Down-regulated in UV response | 1.038232 | 0.857951 | 1.256395 | 0.699824 |
| Apoptosis | 0.965677 | 0.806108 | 1.156833 | 0.704681 |
| Apical surface | 1.036233 | 0.853248 | 1.258462 | 0.719569 |
| DNA repair | 1.031023 | 0.852214 | 1.24735 | 0.753229 |
| Up-regulated in UV response | 0.968229 | 0.78934 | 1.18766 | 0.756725 |
| Peroxisome | 0.979499 | 0.803113 | 1.194624 | 0.837977 |
| Unfolded protein response | 0.982915 | 0.810514 | 1.191988 | 0.860978 |
| Cholesterol homeostasis | 1.014597 | 0.839142 | 1.226737 | 0.881086 |
| Bile acid metabolism | 0.983004 | 0.780963 | 1.237313 | 0.883899 |
| Up-regulated in KRAS signaling | 0.988976 | 0.819332 | 1.193746 | 0.908086 |
| Myogenesis | 1.011335 | 0.833222 | 1.227522 | 0.909212 |
| Xenobiotic metabolism | 0.988329 | 0.785081 | 1.244196 | 0.92039 |
| mTORC1 signaling | 1.007151 | 0.821729 | 1.234414 | 0.945277 |

Table S2: Gene set enrichment analysis of significantly enriched hallmarks in responders in the training cohort.

| ID | Enrichment Score | NES | pvalue | p.adjust | qvalues |
| --- | --- | --- | --- | --- | --- |
| HALLMARK_INTERFERON_GAMMA_RESPONSE | 0.58125761 | 1.72807556 | 0.00011089 | 0.0008731 | 0.00031248 |
| HALLMARK_MYC_TARGETS_V1 | 0.68554881 | 2.03599639 | 0.00011098 | 0.0008731 | 0.00031248 |
| HALLMARK_E2F_TARGETS | 0.7790634 | 2.31409252 | 0.00011107 | 0.0008731 | 0.00031248 |
| HALLMARK_G2M_CHECKPOINT | 0.71346815 | 2.1160995 | 0.00011121 | 0.0008731 | 0.00031248 |
| HALLMARK_OXIDATIVE_PHOSPHORYLATION | 0.61535815 | 1.81952129 | 0.00011188 | 0.0008731 | 0.00031248 |
| HALLMARK_DNA_REPAIR | 0.63833324 | 1.85305163 | 0.0001153 | 0.0008731 | 0.00031248 |
| HALLMARK_INTERFERON_ALPHA_RESPONSE | 0.70175684 | 1.95138417 | 0.00012223 | 0.0008731 | 0.00031248 |
| HALLMARK_MITOTIC_SPINDLE | 0.5083367 | 1.51128212 | 0.00022178 | 0.00116618 | 0.00041737 |
| HALLMARK_MTORC1_SIGNALING | 0.51185962 | 1.52040326 | 0.00022215 | 0.00116618 | 0.00041737 |
| HALLMARK_SPERMATOGENESIS | 0.57711169 | 1.65931929 | 0.00023324 | 0.00116618 | 0.00041737 |
| HALLMARK_ANGIOGENESIS | -0.7627652 | -2.0996446 | 0.0003723 | 0.00169228 | 0.00060566 |
| HALLMARK_ALLOGRAFT_REJECTION | 0.5009181 | 1.4879031 | 0.0004443 | 0.00185123 | 0.00066255 |
| HALLMARK_COAGULATION | -0.6323929 | -2.174595 | 0.00071225 | 0.00220926 | 0.00079068 |
| HALLMARK_UV_RESPONSE_DN | -0.6196321 | -2.1322397 | 0.00071378 | 0.00220926 | 0.00079068 |
| HALLMARK_HYPOXIA | -0.5949678 | -2.1068963 | 0.001 | 0.00220926 | 0.00079068 |
| HALLMARK_ESTROGEN_RESPONSE_EARLY | -0.4191239 | -1.4876907 | 0.001001 | 0.00220926 | 0.00079068 |
| HALLMARK_APICAL_JUNCTION | -0.5170923 | -1.8334736 | 0.00100604 | 0.00220926 | 0.00079068 |
| HALLMARK_KRAS_SIGNALING_DN | -0.4395408 | -1.5584962 | 0.00100604 | 0.00220926 | 0.00079068 |
| HALLMARK_KRAS_SIGNALING_UP | -0.5317326 | -1.8853842 | 0.00100604 | 0.00220926 | 0.00079068 |
| HALLMARK_P53_PATHWAY | -0.4431346 | -1.5699511 | 0.00100908 | 0.00220926 | 0.00079068 |
| HALLMARK_EPITHELIAL_MESENCHYMAL_TRANSITION | -0.8281518 | -2.9451359 | 0.0010101 | 0.00220926 | 0.00079068 |
| HALLMARK_MYOGENESIS | -0.5605696 | -1.9929168 | 0.00101626 | 0.00220926 | 0.00079068 |
| HALLMARK_TNFA_SIGNALING_VIA_NFKB | -0.6512837 | -2.3154202 | 0.00101626 | 0.00220926 | 0.00079068 |
| HALLMARK_TGF_BETA_SIGNALING | -0.5776942 | -1.7224437 | 0.00127823 | 0.00266297 | 0.00095306 |
| HALLMARK_UNFOLDED_PROTEIN_RESPONSE | 0.5360865 | 1.50557067 | 0.00338042 | 0.00676084 | 0.00241967 |
| HALLMARK_ESTROGEN_RESPONSE_LATE | -0.3892826 | -1.3835139 | 0.00603015 | 0.01129178 | 0.00404127 |
| HALLMARK_INFLAMMATORY_RESPONSE | -0.3957641 | -1.4070062 | 0.00609756 | 0.01129178 | 0.00404127 |
| HALLMARK_IL2_STAT5_SIGNALING | -0.3841305 | -1.3634809 | 0.00700701 | 0.01251251 | 0.00447816 |
| HALLMARK_MYC_TARGETS_V2 | 0.58001574 | 1.51201021 | 0.00815428 | 0.01405911 | 0.00503168 |
| HALLMARK_PROTEIN_SECRETION | 0.49721592 | 1.38261465 | 0.02029092 | 0.0338182 | 0.01210335 |
| HALLMARK_APOPTOSIS | -0.3625781 | -1.2634785 | 0.02887789 | 0.04657724 | 0.01666975 |
| HALLMARK_WNT_BETA_CATENIN_SIGNALING | -0.5110499 | -1.452127 | 0.03002766 | 0.04691821 | 0.01679178 |
| HALLMARK_FATTY_ACID_METABOLISM | 0.43488066 | 1.26938254 | 0.04890561 | 0.07409941 | 0.02651979 |
| HALLMARK_PI3K_AKT_MTOR_SIGNALING | 0.46278056 | 1.29739327 | 0.05346559 | 0.07862586 | 0.02813978 |
| HALLMARK_REACTIVE_OXYGEN_SPECIES_PATHWAY | 0.5298355 | 1.33769242 | 0.0695109 | 0.09930128 | 0.03553941 |
| HALLMARK_NOTCH_SIGNALING | -0.5004712 | -1.344181 | 0.07946782 | 0.11037197 | 0.03950155 |
| HALLMARK_COMPLEMENT | -0.3219987 | -1.1437912 | 0.10330579 | 0.13926646 | 0.04984273 |
| HALLMARK_PEROXISOME | 0.4373897 | 1.22621064 | 0.10584251 | 0.13926646 | 0.04984273 |
| HALLMARK_HEDGEHOG_SIGNALING | -0.4603866 | -1.2598098 | 0.12131268 | 0.15552908 | 0.05566304 |
| HALLMARK_XENOBIOTIC_METABOLISM | -0.308812 | -1.0982207 | 0.16161616 | 0.2020202 | 0.07230197 |
| HALLMARK_UV_RESPONSE_UP | 0.39415819 | 1.1492735 | 0.17515996 | 0.21360971 | 0.07644979 |
| HALLMARK_APICAL_SURFACE | -0.4011584 | -1.147835 | 0.20425868 | 0.24316509 | 0.08702751 |
| HALLMARK_ADIPOGENESIS | 0.34957824 | 1.03787917 | 0.38713619 | 0.45015836 | 0.16110931 |
| HALLMARK_IL6_JAK_STAT3_SIGNALING | 0.37361405 | 1.02683183 | 0.41652217 | 0.47332065 | 0.16939897 |
| HALLMARK_GLYCOLYSIS | 0.34171561 | 1.01550883 | 0.44107856 | 0.49008729 | 0.17539966 |
| HALLMARK_ANDROGEN_RESPONSE | -0.2997201 | -0.9798082 | 0.49062845 | 0.53329179 | 0.19086232 |
| HALLMARK_BILE_ACID_METABOLISM | 0.31658633 | 0.89521964 | 0.7018574 | 0.74665681 | 0.26722454 |
| HALLMARK_CHOLESTEROL_HOMEOSTASIS | 0.31939887 | 0.85959055 | 0.74102079 | 0.77189666 | 0.27625775 |
| HALLMARK_PANCREAS_BETA_CELLS | -0.2820949 | -0.7965469 | 0.86333973 | 0.88095891 | 0.31529056 |
| HALLMARK_HEME_METABOLISM | 0.27817483 | 0.82618025 | 0.88610124 | 0.88610124 | 0.31713097 |

Table S3: Univariate Cox analysis of 50 cancer hallmarks in the validation I cohort.

| ID | HR | HR.95L | HR.95H | *P* value |
| --- | --- | --- | --- | --- |
| IFN-α response | 0.640279 | 0.465124 | 0.881391 | 0.006253 |
| Apoptosis | 0.666838 | 0.49531 | 0.897766 | 0.007567 |
| Complement | 0.701181 | 0.524803 | 0.936837 | 0.016336 |
| Allograft rejection | 0.771355 | 0.588309 | 1.011352 | 0.060341 |
| Inflammatory response | 0.761207 | 0.571653 | 1.013615 | 0.061845 |
| IL2/STAT5 signaling | 0.783525 | 0.599393 | 1.024222 | 0.074284 |
| IFN-γ response | 0.774077 | 0.584074 | 1.025889 | 0.074734 |
| TNF-α signaling via NF-κB | 0.770401 | 0.57777 | 1.027257 | 0.075604 |
| IL6/JAK/STAT3 signaling | 0.785622 | 0.592818 | 1.041132 | 0.093074 |
| Up-regulated in KRAS signaling | 0.804856 | 0.607063 | 1.067095 | 0.131384 |
| Unfolded protein response | 0.806277 | 0.599049 | 1.085191 | 0.155435 |
| Androgen response | 0.837339 | 0.629407 | 1.113963 | 0.222869 |
| Up-regulated in UV response | 0.827835 | 0.609822 | 1.12379 | 0.225672 |
| Bile acid metabolism | 1.188917 | 0.890296 | 1.587702 | 0.24097 |
| Pancreatic β cells | 0.866683 | 0.657989 | 1.141568 | 0.308693 |
| Peroxisome | 1.156224 | 0.873005 | 1.531326 | 0.311262 |
| Adipogenesis | 1.163915 | 0.86522 | 1.565727 | 0.315778 |
| TGF-β signaling | 0.871483 | 0.665074 | 1.141953 | 0.318544 |
| Notch signaling | 1.204434 | 0.823926 | 1.760667 | 0.336955 |
| mTORC1 signaling | 0.863332 | 0.63704 | 1.170007 | 0.343348 |
| Protein secretion | 0.88527 | 0.672608 | 1.165171 | 0.384638 |
| MYC targets v1 | 0.885043 | 0.664832 | 1.178194 | 0.402825 |
| Wnt/β-Catenin signaling | 1.124794 | 0.853437 | 1.48243 | 0.403795 |
| MYC targets v2 | 0.890996 | 0.671155 | 1.182847 | 0.424654 |
| Spermatogenesis | 1.131275 | 0.835116 | 1.532461 | 0.425759 |
| PI3K/AKT/mTOR signaling | 0.925559 | 0.717213 | 1.194428 | 0.552165 |
| Down-regulated in UV response | 0.917742 | 0.68893 | 1.222548 | 0.557428 |
| Down-regulated in KRAS signaling | 1.073706 | 0.821179 | 1.40389 | 0.603175 |
| Early estrogen response | 0.927917 | 0.683404 | 1.259913 | 0.631644 |
| P53 pathway | 0.937121 | 0.70512 | 1.245457 | 0.654524 |
| Glycolysis | 1.067635 | 0.789108 | 1.444471 | 0.671331 |
| Myogenesis | 1.062306 | 0.798458 | 1.413342 | 0.678206 |
| Apical junction | 0.944361 | 0.713968 | 1.249101 | 0.68828 |
| Angiogenesis | 0.942684 | 0.705065 | 1.260385 | 0.690405 |
| Hedgehog signaling | 1.057218 | 0.795137 | 1.405684 | 0.701863 |
| Coagulation | 0.9465 | 0.710745 | 1.260455 | 0.706762 |
| Heme metabolism | 0.946706 | 0.709073 | 1.263977 | 0.710352 |
| Hypoxia | 0.948655 | 0.70733 | 1.272315 | 0.724886 |
| DNA repair | 1.054851 | 0.775928 | 1.434037 | 0.733248 |
| Apical surface | 0.952532 | 0.718661 | 1.262512 | 0.735125 |
| Late estrogen response | 0.954152 | 0.699027 | 1.302391 | 0.767502 |
| Cholesterol homeostasis | 0.962054 | 0.700979 | 1.320366 | 0.810728 |
| Xenobiotic metabolism | 0.963181 | 0.705507 | 1.314966 | 0.8133 |
| Mitotic spindle | 0.978893 | 0.727423 | 1.317296 | 0.888013 |
| Fatty acid metabolism | 0.98303 | 0.725711 | 1.331589 | 0.911987 |
| Oxidative phosphorylation | 1.010092 | 0.75391 | 1.353327 | 0.946358 |
| Epithelial-mesenchymal transition | 0.992854 | 0.748072 | 1.317734 | 0.9604 |
| Reactive oxygen species pathway | 0.99561 | 0.730862 | 1.35626 | 0.977746 |
| G2M checkpoint | 0.996336 | 0.73266 | 1.354905 | 0.981327 |
| E2F targets | 1.00057 | 0.737339 | 1.357776 | 0.997081 |

Table S4: Gene set enrichment analysis of significantly enriched hallmarks in responders in the validation I cohort.

| ID | Enrichment Score | NES | pvalue | p.adjust | qvalues |
| --- | --- | --- | --- | --- | --- |
| HALLMARK_E2F_TARGETS | 0.67542867 | 3.09617051 | 0.0006402 | 0.00222578 | 0.00112461 |
| HALLMARK_INTERFERON_ALPHA_RESPONSE | 0.73723485 | 3.04862327 | 0.00043898 | 0.00199537 | 0.00100819 |
| HALLMARK_EPITHELIAL_MESENCHYMAL_TRANSITION | -0.7611788 | -2.9401979 | 0.00011723 | 0.00082598 | 0.00041734 |
| HALLMARK_G2M_CHECKPOINT | 0.632016 | 2.89716648 | 0.0006402 | 0.00222578 | 0.00112461 |
| HALLMARK_INTERFERON_GAMMA_RESPONSE | 0.55190744 | 2.53718808 | 0.00064516 | 0.00222578 | 0.00112461 |
| HALLMARK_MYC_TARGETS_V1 | 0.52055287 | 2.42805548 | 0.00068027 | 0.00222578 | 0.00112461 |
| HALLMARK_MYOGENESIS | -0.6345914 | -2.2971715 | 0.0001256 | 0.00082598 | 0.00041734 |
| HALLMARK_MYC_TARGETS_V2 | 0.60373808 | 2.22427275 | 0.00036049 | 0.00180245 | 0.00091071 |
| HALLMARK_COAGULATION | -0.6487297 | -2.1942316 | 0.00013268 | 0.00082598 | 0.00041734 |
| HALLMARK_HYPOXIA | -0.5500034 | -2.0674663 | 0.00012117 | 0.00082598 | 0.00041734 |
| HALLMARK_ANGIOGENESIS | -0.7231188 | -2.0550476 | 0.00014868 | 0.00082598 | 0.00041734 |
| HALLMARK_UV_RESPONSE_DN | -0.5344557 | -1.9815808 | 0.00012277 | 0.00082598 | 0.00041734 |
| HALLMARK_SPERMATOGENESIS | 0.5214415 | 1.96356098 | 0.00110579 | 0.00290997 | 0.0014703 |
| HALLMARK_TNFA_SIGNALING_VIA_NFKB | -0.5068871 | -1.9410383 | 0.00011848 | 0.00082598 | 0.00041734 |
| HALLMARK_APICAL_JUNCTION | -0.5062107 | -1.900459 | 0.00012129 | 0.00082598 | 0.00041734 |
| HALLMARK_TGF_BETA_SIGNALING | -0.5686839 | -1.8346426 | 0.00096246 | 0.00276706 | 0.00139809 |
| HALLMARK_P53_PATHWAY | -0.473714 | -1.8186419 | 0.00011827 | 0.00082598 | 0.00041734 |
| HALLMARK_MITOTIC_SPINDLE | 0.3791183 | 1.77721788 | 0.00071225 | 0.00222578 | 0.00112461 |
| HALLMARK_KRAS_SIGNALING_UP | -0.4661554 | -1.7053813 | 0.00099614 | 0.00276706 | 0.00139809 |
| HALLMARK_NOTCH_SIGNALING | -0.5959555 | -1.6475653 | 0.01108448 | 0.02639162 | 0.01333471 |
| HALLMARK_WNT_BETA_CATENIN_SIGNALING | -0.5593171 | -1.6156566 | 0.01340009 | 0.03045475 | 0.01538766 |
| HALLMARK_APICAL_SURFACE | -0.5510986 | -1.5398614 | 0.02696225 | 0.05617136 | 0.02838132 |
| HALLMARK_ADIPOGENESIS | -0.3849347 | -1.4778079 | 0.00626848 | 0.0156712 | 0.00791808 |
| HALLMARK_DNA_REPAIR | 0.32760234 | 1.41944218 | 0.01465387 | 0.03185623 | 0.01609578 |
| HALLMARK_HEDGEHOG_SIGNALING | -0.5468712 | -1.4010772 | 0.08292988 | 0.13375787 | 0.06758292 |
| HALLMARK_BILE_ACID_METABOLISM | -0.421271 | -1.3817935 | 0.06211688 | 0.110923 | 0.0560453 |
| HALLMARK_ESTROGEN_RESPONSE_EARLY | -0.3606278 | -1.3666009 | 0.03238575 | 0.0647715 | 0.03272665 |
| HALLMARK_ALLOGRAFT_REJECTION | 0.31298841 | 1.33380221 | 0.03892359 | 0.07485307 | 0.0378205 |
| HALLMARK_ESTROGEN_RESPONSE_LATE | -0.3531766 | -1.3196018 | 0.05837996 | 0.10811103 | 0.05462452 |
| HALLMARK_XENOBIOTIC_METABOLISM | -0.3616559 | -1.3188426 | 0.0673089 | 0.11604982 | 0.0586357 |
| HALLMARK_GLYCOLYSIS | -0.3368212 | -1.2734756 | 0.08186853 | 0.13375787 | 0.06758292 |
| HALLMARK_HEME_METABOLISM | -0.3238874 | -1.210166 | 0.14242277 | 0.21579207 | 0.10903178 |
| HALLMARK_KRAS_SIGNALING_DN | -0.3738835 | -1.2061931 | 0.19441771 | 0.26754674 | 0.13518151 |
| HALLMARK_ANDROGEN_RESPONSE | -0.3393155 | -1.2032817 | 0.17396328 | 0.24851897 | 0.12556748 |
| HALLMARK_IL2_STAT5_SIGNALING | -0.3204837 | -1.2031871 | 0.14942389 | 0.21974102 | 0.11102704 |
| HALLMARK_UV_RESPONSE_UP | 0.27426932 | 1.17838103 | 0.14100648 | 0.21579207 | 0.10903178 |
| HALLMARK_REACTIVE_OXYGEN_SPECIES_PATHWAY | -0.3884328 | -1.1592001 | 0.25731917 | 0.32164897 | 0.16251737 |
| HALLMARK_IL6_JAK_STAT3_SIGNALING | 0.30778656 | 1.1590134 | 0.20493918 | 0.26965682 | 0.13624766 |
| HALLMARK_COMPLEMENT | -0.2995355 | -1.1191782 | 0.25711506 | 0.32164897 | 0.16251737 |
| HALLMARK_INFLAMMATORY_RESPONSE | -0.3059314 | -1.1177497 | 0.26849315 | 0.32743067 | 0.16543866 |
| HALLMARK_OXIDATIVE_PHOSPHORYLATION | 0.24699264 | 1.11011738 | 0.19798459 | 0.26754674 | 0.13518151 |
| HALLMARK_APOPTOSIS | -0.2921173 | -1.0867894 | 0.30848329 | 0.36724201 | 0.18555386 |
| HALLMARK_FATTY_ACID_METABOLISM | 0.23940449 | 1.02123012 | 0.41029341 | 0.47708536 | 0.24105366 |
| HALLMARK_CHOLESTEROL_HOMEOSTASIS | -0.2918937 | -0.9627072 | 0.52218754 | 0.59339493 | 0.2998206 |
| HALLMARK_PANCREAS_BETA_CELLS | -0.399406 | -0.9337683 | 0.55843735 | 0.62048594 | 0.31350869 |
| HALLMARK_MTORC1_SIGNALING | -0.2153603 | -0.827597 | 0.82666351 | 0.89854729 | 0.45400284 |
| HALLMARK_PROTEIN_SECRETION | -0.2188937 | -0.7762416 | 0.86211324 | 0.91426665 | 0.46194525 |
| HALLMARK_PI3K_AKT_MTOR_SIGNALING | -0.2162249 | -0.7613154 | 0.87769598 | 0.91426665 | 0.46194525 |
| HALLMARK_UNFOLDED_PROTEIN_RESPONSE | 0.17066851 | 0.72470795 | 0.97057405 | 0.97057405 | 0.49039531 |
| HALLMARK_PEROXISOME | -0.2109072 | -0.7225481 | 0.91342451 | 0.93206583 | 0.47093852 |
